# Prognostic significance and biological implications of *SM*-like genes in mantle cell lymphoma

**DOI:** 10.1007/s44313-024-00037-3

**Published:** 2024-10-17

**Authors:** Xue He, Changjian Yan, Yaru Yang, Weijia Wang, Xiaoni Liu, Chaoling Wu, Zimu Zhou, Xin Huang, Wei Fu, Jing Hu, Ping Yang, Jing Wang, Mingxia Zhu, Yan Liu, Wei Zhang, Shaoxiang Li, Gehong Dong, Xiaoliang Yuan, Yuansheng Lin, Hongmei Jing, Weilong Zhang

**Affiliations:** 1 Department of Pathology, Beijing Tiantan Hospital, Capital Medical University, Beijing, 100070 China; 2 Department of Hematology, Lymphoma Research Center, Peking University Third Hospital, Beijing, 100191 China; 3 The Second Affiliated Hospital of Chongqing Medical University, Chongqing, 400016 China; 4 Department of Pathology, The First Affiliated Hospital of Nanchang University, Nanchang, 330006 China; 5 Department of Respiratory Medicine, The First Affiliated Hospital of Gannan Medical University, Ganzhou, 341000 China; 6 Department of Respiratory Medicine, Affiliated Hospital of Jiujiang University, Jiujiang, 332000 China; 7 Gannan Medical University, Ganzhou, 341000 China; 8 The Second Affiliated Hospital of Fujian Medical University, Quanzhou, 362000 China; 9 Department of Intensive Care Unit, Suzhou Research Center of Medical School, Suzhou Hospital, Affiliated Hospital of Medical School, Nanjing University, Suzhou, 215000 China

**Keywords:** *LSM* genes, Mantle cell lymphoma, RNA degradation, *LSM1*, *LSM8*

## Abstract

**Background:**

SM-like (*LSM*) genes a family of RNA-binding proteins, are involved in mRNA regulation and can function as oncogenes by altering mRNA stability. However, their roles in B-cell progression and tumorigenesis remain poorly understood.

**Methods:**

We analyzed gene expression profiles and overall survival data of 123 patients with mantle cell lymphoma (MCL). The LSM index was developed to assess its potential as a prognostic marker of MCL survival.

**Results:**

Five of the eight *LSM* genes were identified as potential prognostic markers for survival in MCL, with particular emphasis on the *LSM*.index. The expression levels of these *LSM* genes demonstrated their potential utility as classifiers of MCL. The *LSM*.index-high group exhibited both poorer survival rates and lower RNA levels than did the overall transcript profile. Notably, *LSM1* and *LSM8* were overexpressed in the *LSM*.index-high group, with *LSM1* showing 2.5-fold increase (*p* < 0.001) and *LSM8* depicting 1.8-fold increase (*p* < 0.01) than those in the *LSM*.index-low group. Furthermore, elevated *LSM* gene expression was associated with increased cell division and RNA splicing pathway activity.

**Conclusions:**

The *LSM*.index demonstrates potential as a prognostic marker for survival in patients with MCL. Elevated expression of *LSM* genes, particularly *LSM1* and *LSM8*, may be linked to poor survival outcomes through their involvement in cell division and RNA splicing pathways. These findings suggest that *LSM* genes may contribute to the aggressive behavior of MCL and represent potential targets for therapeutic interventions.

**Supplementary Information:**

The online version contains supplementary material available at 10.1007/s44313-024-00037-3.

## Introduction

RNA degradation is a conserved and ubiquitous process in all cells critical for the proper regulation of genetic information [1]. In eukaryotic cells, mRNA degradation occurs primarily via two pathways: the 5′ to 3′ pathway and the 3′ to 5′ pathway [1]. The cytoplasmic *LSM1-7* complex (comprising *LSM1–LSM7*) and the nuclear *LSM2–8* complex (comprising *LSM2–LSM8*) are distinct, conserved heptamer complexes that facilitate the 5' to 3' RNA degradation pathway [2–7]. The subunits *LSM1* and *LSM8* are critical for distinguishing the two complexes: the *LSM1-7* complex (cytoplasmic, involved in mRNA degradation but not for RNA splicing) and the *LSM2-8* complex (nuclear, involved in pre-mRNA degradation and necessary for RNA splicing) [2–7]. The mRNA decay mediated by the *LSM1–7* complex in the cytoplasm and the pre-mRNA decay mediated by the *LSM2–8* complex in the nucleus share several similarities. In both complexes, deadenylation initiates mRNA decay [8, 9], followed by decapping and degradation via the 5′ to 3′ exonucleolytic pathway [10, 11]. In the *LSM1–7* complex-mediated pathway, this complex promotes decapping by interacting with decapping activators such as Dhh1 and Pat1 [12, 13]. The mRNA decay rate is slower in *LSM1–7* mutants [14].

Mantle cell lymphoma (MCL) is a subtype of non-Hodgkin B cell lymphoma that primarily affects older individuals [15, 16]. Although chemotherapy and stem cell transplantation have improved the prognosis, MCL still has a short median survival of approximately 5 to 7 years due to its aggressive behavior and rapid progression [17]. Understanding the molecular mechanisms driving MCL’s aggressive nature and identifying new treatment strategies are crucial. *LSM1* overexpression has been observed in both primary and metastatic tumors [18, 19]. In human breast cancer, the chromosome region 8p11-12 containing *LSM1* gene is frequently amplified [20, 21]. However, little is known about the prognostic significance and biological implications of *LSM* family genes in MCL. In this study, we demonstrated the prognostic relevance and biological implications of *LSM* genes in MCL.

## Methods

### Data source

We obtained 123 MCL gene expression arrays (Affymetrix Human Genome U133 Plus 2.0 Array) from the NCBI Gene Expression Omnibus (GEO) database (GSE93291) [22]. Additionally, 64 MCL gene expression arrays (Affymetrix Human Genome U133 Plus 2.0 Array) were obtained from the NCBI GEO database (GSE21452) [23]. Dataset GSE21452 represents the first phase of dataset GSE93291 [23]. These samples were derived from diagnostic lymph node tissues in formalin-fixed paraffin-embedded biopsies with a tumor content of ≥ 60%. All patients underwent treatment, including R-CHOP therapy, followingdiagnostic biopsy. Notably, dataset GSE21452 represents the first phase of GSE93291 [24]. This study was conducted in accordance with the principles of the Declaration of Helsinki.

### Gene expression analysis

Probeset measures for all arrays (123 MCL samples) were calculated using the Robust Multiarray Averaging algorithm. The relative RNA expression values for each probe were log-transformed (log2). Data comparing the *LSM*.index-high group to the *LSM*.index-low group were analyzed using an unpaired t-test and presented as mean ± SEM. Only genes with a fold change (log2) > 1 or < -1 and *P*-value < 0.05 were considered differentially expressed genes.

### Definition of *LSM*.index for survival prediction

A comprehensive *LSM*.index was defined to predict survival in patients with MCL. The *LSM*.index was calculated using Eq. (1).

1 $$LSM{.index}_{j}= {H}_{j }/{ F}_{j}$$

Where *LSM.index*_*j*_ indicates the index of *LSM* genes of j^th^ sample used in survival prediction.

*H*_j_ indicates the product of the expression of harmful genes with a *P*-value < 0.05 in the j^th^ sample. Three of the eight *LSM* genes (*LSM1*, *LSM2*, and *LSM4*) with a hazard ratio of > 1 were included.

*F*_j_ indicates the product of favorable gene expression in the j^th^ sample. One of the eight *LSM* genes (*LSM8*) with a hazard ratio of < 1 was included.

### Gene Ontology (GO) analysis

Pathway enrichment analysis for differentially expressed genes between the LSM.index-high and *LSM*.index-low groups in MCL was conducted using the DAVID tool with default parameters [25]. The enriched GO pathway terms shown in the main figure were manually curated by selecting nonredundant GO terms from the biological process category.

### Statistics

R software v3.1.3, with the ggplot2 and survminer packages, was used for statistical analysis. Data were expressed as mean ± SEM in bar plots. A *P*-value < 0.05 was considered statistically significant.

## Results

### Five of eight *LSM* genes predict survival in MCL

**Fig. 1:**
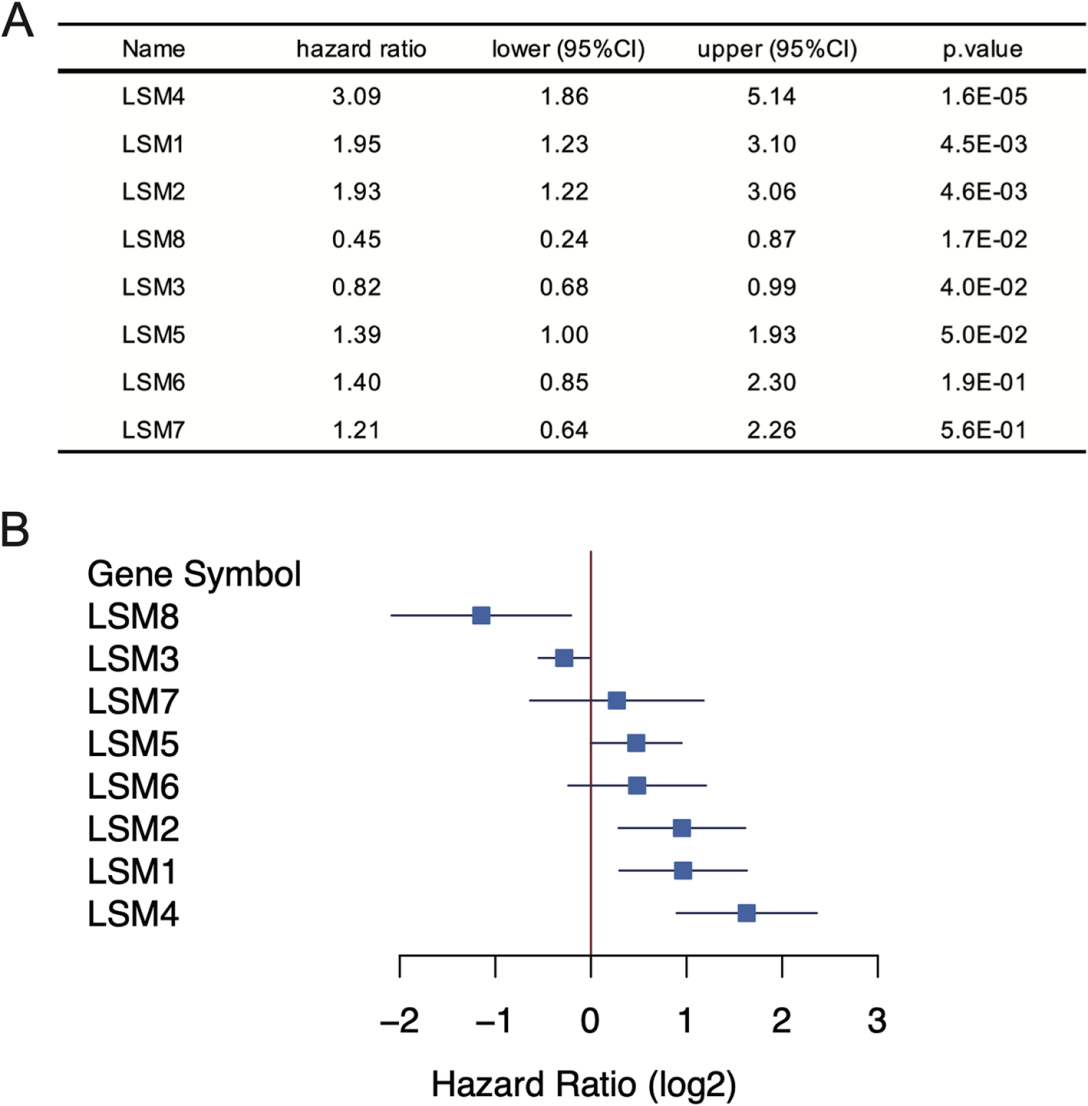
Eight *LSM* genes survival analysis and forest plot of 123 MCL patients. **A** Eight *LSM* genes survival analysis (sorted by *P*-value). **B** Forest plot of eight *LSM* genes (sorted by hazard ratio). The forest plot shows the lower and upper 95%CI (confidence interval)

**Fig. 2:**
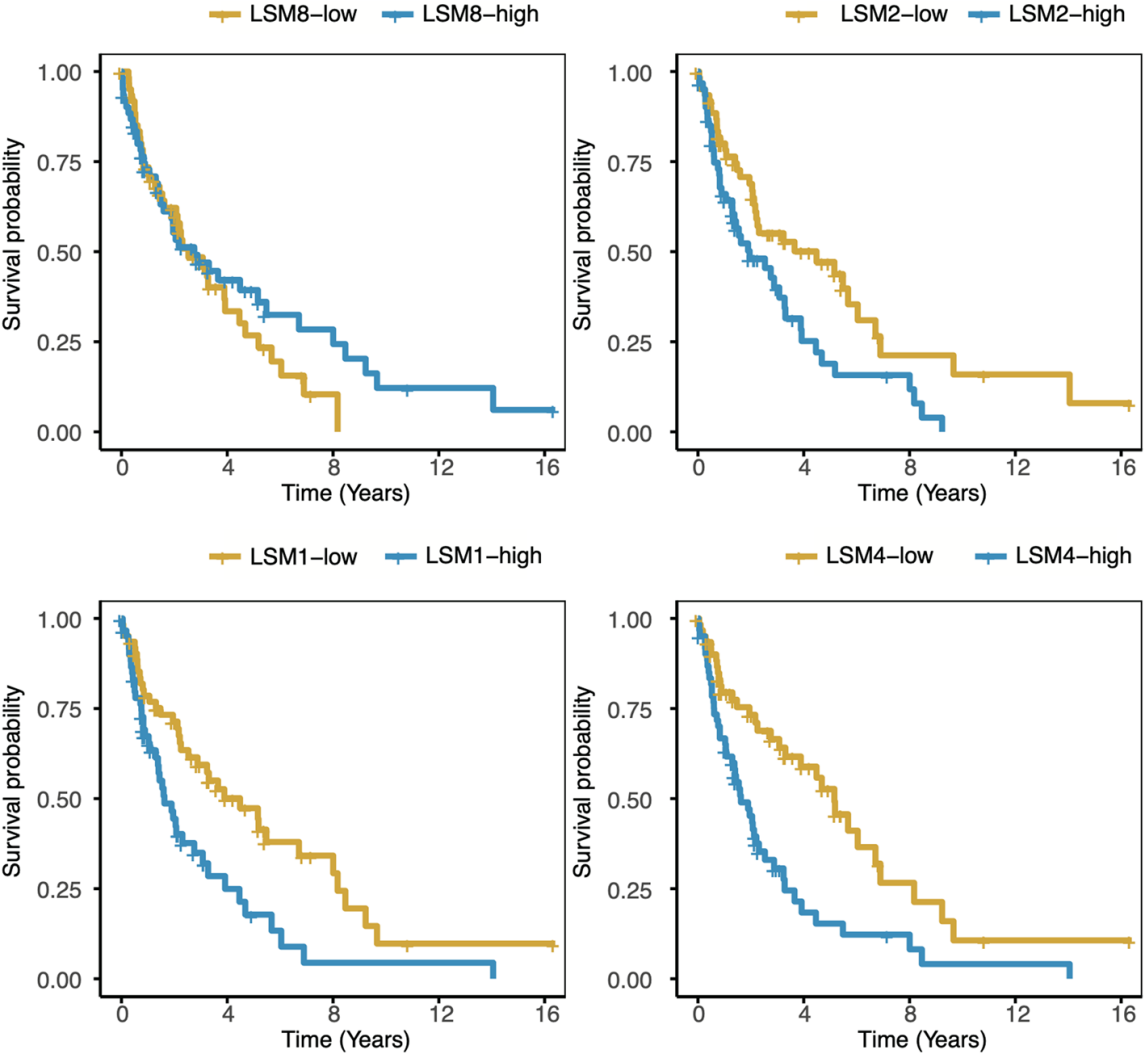
Overall survival of 123 MCL patients and Kaplan–Meier curves of four *LSM* genes. *LSM8*, *P* = 1.7E-02; *LSM2*, *P* = 4.6E-03; *LSM1*, *P* = 4.5E-03; *LSM4*, *P* = 1.6E-05. To compare Kaplan–Meier curves, a log-rank test was used

To investigate the relationship between the *LSM* genes (*LSM1*, *LSM2*, *LSM3*, *LSM4*, *LSM5*, *LSM6*, *LSM7*, and *LSM8*) and survival in MCL, we analyzed the expression profiles of 123 MCL samples from the GSE21452 dataset. The expression levels of five out of the eight *LSM* genes were significantly associated with MCL survival (*p* < 0.05, log-rank test). The eight *LSM* genes were classified based on their hazard ratio values. Two of the *LSM* genes (*LSM3*, *LSM8*) had a hazard ratio of less than 1 and were defined as “favorable genes,” which are considered beneficial for MCL survival. Among these, *LSM8*, with a hazard ratio of 0.45 (95%CI, 0.24–0.87), was the most significant among the “favorable genes.” The other six LSM genes (*LSM1*, *LSM2*, *LSM4*, *LSM5*, *LSM6*, and *LSM7*) had a hazard ratio greater than 1 and were classified as “harmful genes,” which are considered detrimental to MCL survival (Fig. 1). *LSM4*, with a hazard ratio of 3.09 (95%CI, 1.35–4.48), was the most significant among the “harmful genes.” Furthermore, Kaplan–Meier survival curves for the four *LSM* genes were compared for 123 patients with MCL using the log-rank test (Fig. 2; *LSM8*, *P* = 1.7E-02; *LSM2*, *P* = 4.6E-03; *LSM1*, *P* = 4.5E-03; *LSM4*, *P* = 1.6E-05, log-rank test). Both the “favorable” and “harmful” *LSM* genes showed significant associations with MCL survival predictions.

We also compared 123 MCL samples from the GSE93291 dataset with 12 reactive lymph node samples from the GSE78513 dataset. We found that in the reactive lymph node group, the expression levels of *LSM4*, *LSM8*, and *LSM12* were higher, whereas in the MCL group, *LSM2*, *LSM5*, *LSM6*, *LSM7*, and *LSM14B* were highly expressed (all *p* < 0.05; Supplementary Fig. 1A). No significant differences were observed in the expression of the remaining genes. Although *LSM1* was identified as a key differentiator between prognostic groups (*P* = 4.5E-03, Fig. 2), there was no significant difference in its expression levels between MCL and reactive lymph nodes (*p* < 0.05, Supplementary Fig. 1A), contrary to *LSM8*. This finding suggests that although *LSM1* may play a role in the biological differences between prognostic groups, it may not be a specific biomarker for distinguishing between MCL and reactive lymph nodes.

### Correlation of 8 *LSM* gene expressions in MCL

**Fig. 3:**
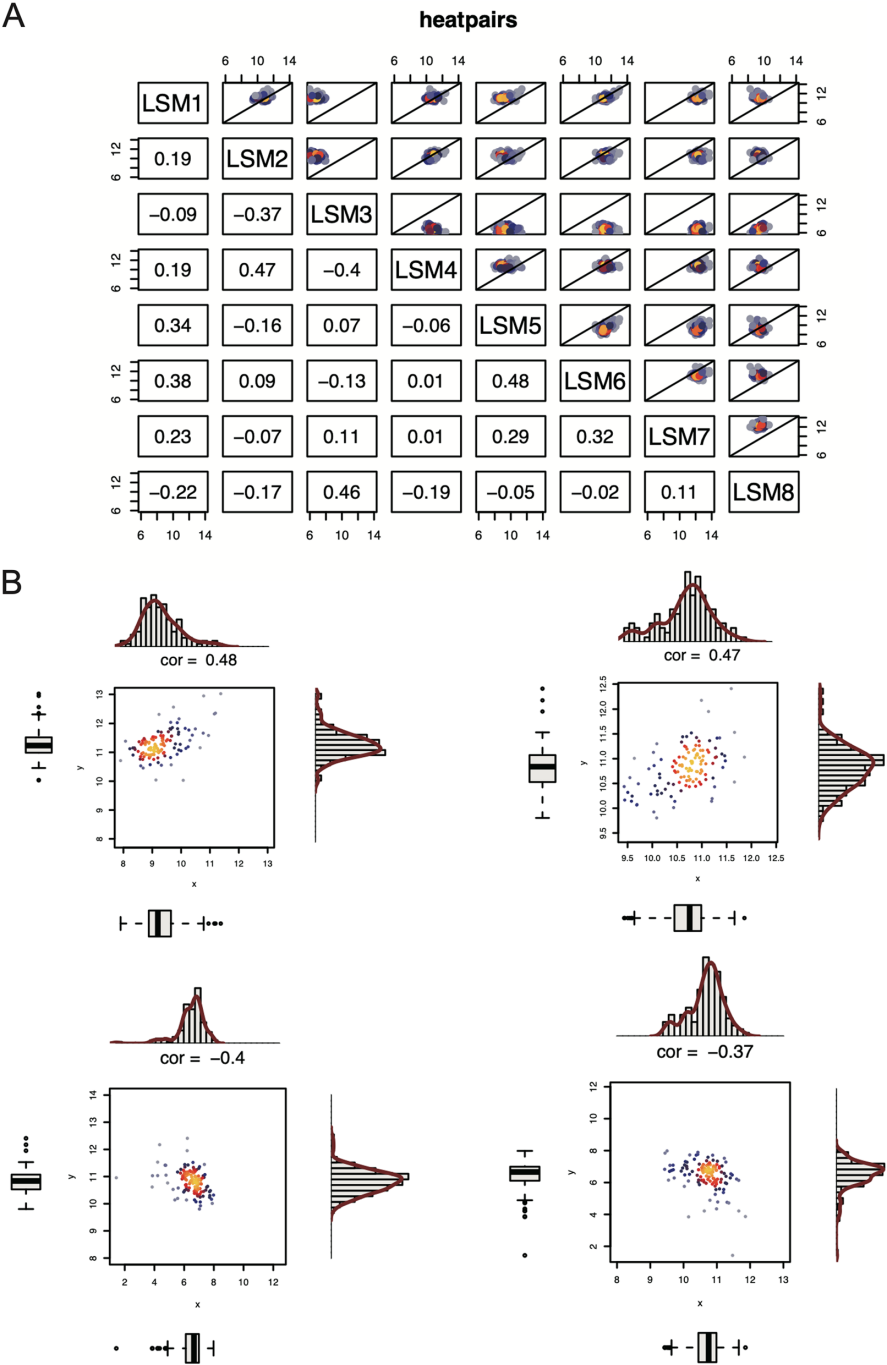
Correlation plot of eight *LSM* gene expression levels in 123 MCL samples. **A** Correlation plot of eight *LSM* gene expressions. The correlation coefficient (cor) is shown in the plot (Pearson correlation). The X-axis and Y-axis are gene expression levels (log2). **B** Correlation of *LSM5* and *LSM6* (left up), *LSM2* and *LSM4* (right up), *LSM3* and *LSM4* (left down), *LSM2* and *LSM3* (right down)

A correlation plot of the expression levels of the eight *LSM* genes in the MCL is shown in Fig. 3. Considerably, some *LSM* genes exhibited positive correlations, including *LSM5* and *LSM6* (correlation coefficient, cor = 0.48), *LSM2* and *LSM4* (cor = 0.47), *LSM3* and *LSM8* (cor = 0.46), and *LSM1* and *LSM6* (cor = 0.38). Conversely, some *LSM* genes displayed negative correlations, such as *LSM3* and *LSM4* (cor = -0.4) and *LSM2* and *LSM3* (cor = -0.37). Additionally, some *LSM* genes, such as *LSM4* and *LSM7*, showed no correlation (cor = 0.01). The “favorable genes” *LSM3* and *LSM8*, which are associated with better survival outcomes in MCL, exhibited a positive correlation (cor = 0.46). In contrast, the “harmful gene” *LSM4*, which is associated with poorer survival outcomes, was negatively correlated (cor = -0.4) with the “favorable genes” *LSM3*.

### Expression of *LSM* genes predicts survival in MCL

**Fig. 4:**
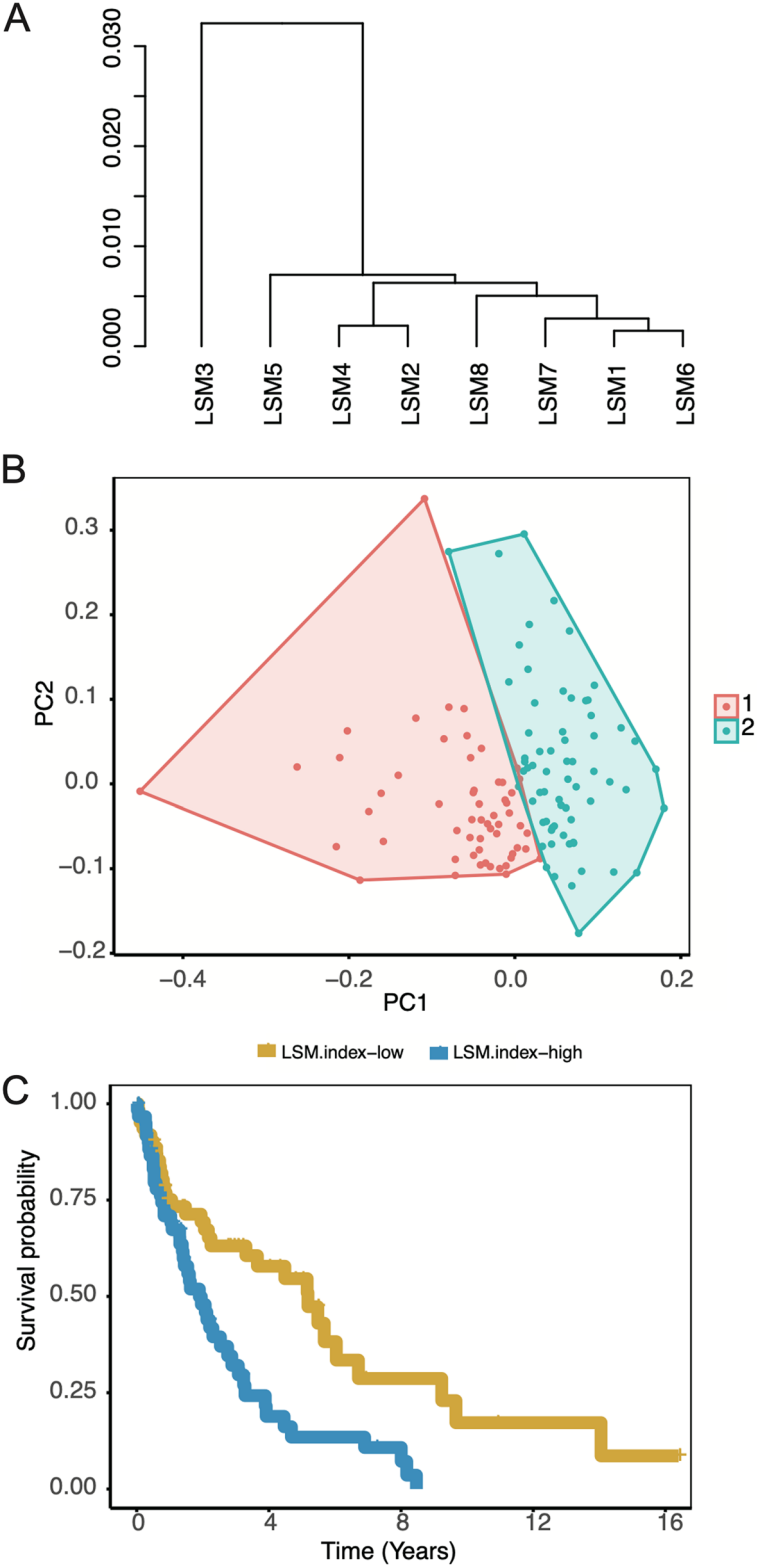
In the 123 MCL patients, eight *LSM* genes were used as a classifier. **A** Unsupervised clustering of eight *LSM* genes expression in 123 MCL patients. Cosine correlation similarity is presented by the cluster of *LSM* genes. **B** The fuzzy clustering of 123 MCL patients through the expression of eight *LSM* genes. **C** Overall survival of 123 MCL patients and Kaplan–Meier curves of the LSM.index (*P* = 3.29E-06). To compare Kaplan–Meier curves, a log-rank test was used

We performed unsupervised clustering of the expression levels of the eight *LSM* genes in 123 patients with MCL using cosine correlation similarity (Fig. 4A). Considerably, the eight *LSM* genes were grouped into two clusters, one containing *LSM3* and the other containing the remaining seven *LSM* genes. Notably, all “harmful genes” were grouped together, suggesting that the expression patterns of LSM genes are linked to MCL survival outcomes and exhibit distinct expression characteristics. Furthermore, we identified that MCL could be categorized into two groups based on fuzzy clustering analysis of the expression of the eight *LSM* genes in 123 MCL samples (Fig. 4B; R, ggplot2). To quantify the imbalance between the expression levels of “harmful” and “favorable” genes, we calculated a ratio termed the *LSM*.index (refer to methods for the definition). The *LSM*.index was strongly associated with MCL survival (Fig. 4C; *P* = 3.29E-06, log-rank test). The hazard ratio for the *LSM*.index was 1.63 (95%CI, 1.33–2.00). A high *LSM*.index was associated with poor survival in patients with MCL, whereas a low *LSM*.index was associated with better survival outcomes.

### *LSM*.index-high group shows low RNA levels in MCL

**Fig. 5:**
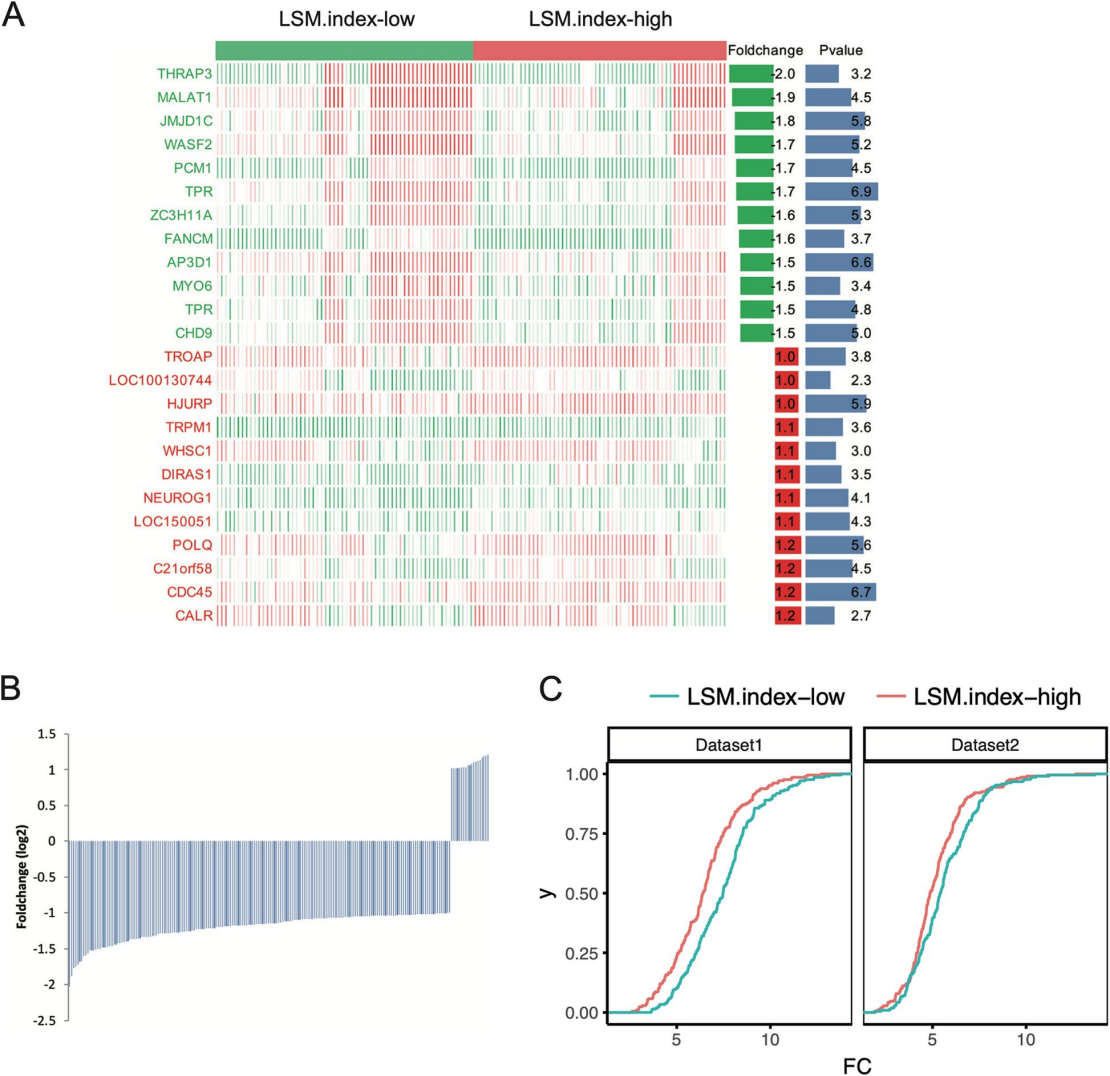
Differential gene expression between *LSM*.index-high and *LSM*.index-low groups in MCL patients. **A** Differential gene expression between the *LSM*.index-high and *LSM*.index-low groups is presented by heatmap. High expression is indicated by red, low expression by green, and intermediate expression by white. The top 12 upregulated and 12 downregulated genes are displayed. The accompanying bar plots represent bar plots represent fold change (log2, left) and *P*-value (-log10, right) in the heatmap. **B** A total of 19 upregulated genes and 190 downregulated genes between *LSM*.index-high and *LSM*.index-low group in MCL. **C** Cumulative distribution of RNA expression levels of different genes between the *LSM*.index-high and *LSM*.index-low in MCL (log2 foldchange). The GSE93291 dataset is presented in the left plot (total 123 samples), whereas the GSE21452 dataset appears in the right plot (total 64 samples)

The *LSM*.index-high and *LSM*.index-low groups represent two distinct classes of MCL. To explore the differences between these groups, we compared their gene expression profiles (Fig. 5A). We identified a total of 19 upregulated and 190 downregulated genes in the *LSM*.index-high group compared to those in the *LSM*. index-low group (Fig. 5B, *p* < 0.05). The *LSM*.index-high group exhibited a higher number of downregulated genes than upregulated genes, suggesting a different RNA metabolism process compared with the *LSM*.index-low group. The cumulative distribution of DEG RNA expression levels for differentially expressed genes between the *LSM*.index-high and *LSM*.index-low groups also showed that the *LSM*.index-high group had lower overall RNA levels (Fig. 5C, *P* = 2.57E-07). This finding was further validated using another dataset (GSE21452, 64 samples,* P* = 0.0048).

We also examined the differences in known prognostic molecular markers for MCL between the high and low *LSM* index groups, along with the differential expression of genes within the *LSM* gene family and their correlation with survival rates. Among the known MCL molecular markers, MKI67 expression was significantly lower in the low *LSM*.index group (*p* = 0.0004, Supplementary Table 1, Supplementary Fig. 1B). Within the *LSM* gene family, *LSM4*, *LSM1*, *LSM6*, and *LSM2* had lower expression in the low *LSM* index group, whereas *LSM8*, *LSM3*, *LSM14A*, and *LSM12* showed higher expression (all *p* < 0.05). No statistically significant differences were observed for *LSM5*, *LSM7*, *LSM14B*, or *LSM10* levels between the two groups. The low *LSM* index group was associated with a longer survival time and lower mortality rate (all *p* < 0.05).

We further investigated differences in MCL35 classification genes between the high- and low-*LSM* index groups. In the low LSM index group, the expression levels of *GLIPR1*, *CHD4*, *GSK3B*, *IK*, and *ATL1* were higher (all *p* < 0.05; Supplementary Fig. 2A). In contrast, the LSM index-high group exhibited elevated expression levels of *UBXN4*, *CDKN3*, *FOXM1*, *H2AFX*, *FAM83D*, *MKI67*, *CDC20*, *NCAPG*, *CCNB2*, *ESPL1*, *KIF2C*, and *ZWINT* (all *p* < 0.05). These differentially expressed genes (*GLIPR1*, *CHD4*, *GSK3B*, *IK*, *ATL1*, *UBXN4*, *CDKN3*, *FOXM1*, *H2AFX*, *FAM83D*, *MKI67*, *CDC20*, *NCAPG*, *CCNB2*, *ESPL1*, *KIF2C*, and *ZWINT*) may represent downstream targets and potential therapeutic targets; however, further investigation and validation are required.

Univariate and multivariate Cox regression analyses were conducted on the *LSM* index, additional *LSM* gene family members (*LSM10*, *LSM11*, *LSM12*, *LSM14A*, and *LSM14B*), *SOX11*, and *TP53*. Variables with *p*-values less than 0.15 were included in the multivariate Cox regression analysis. Our findings indicated that the *LSM* index can serve as an independent prognostic factor (*p* < 0.05; Supplementary Table 2).

### Positive regulation of cell division and RNA splicing pathways are significantly enriched pathways of *LSM* in MCL

**Fig. 6:**
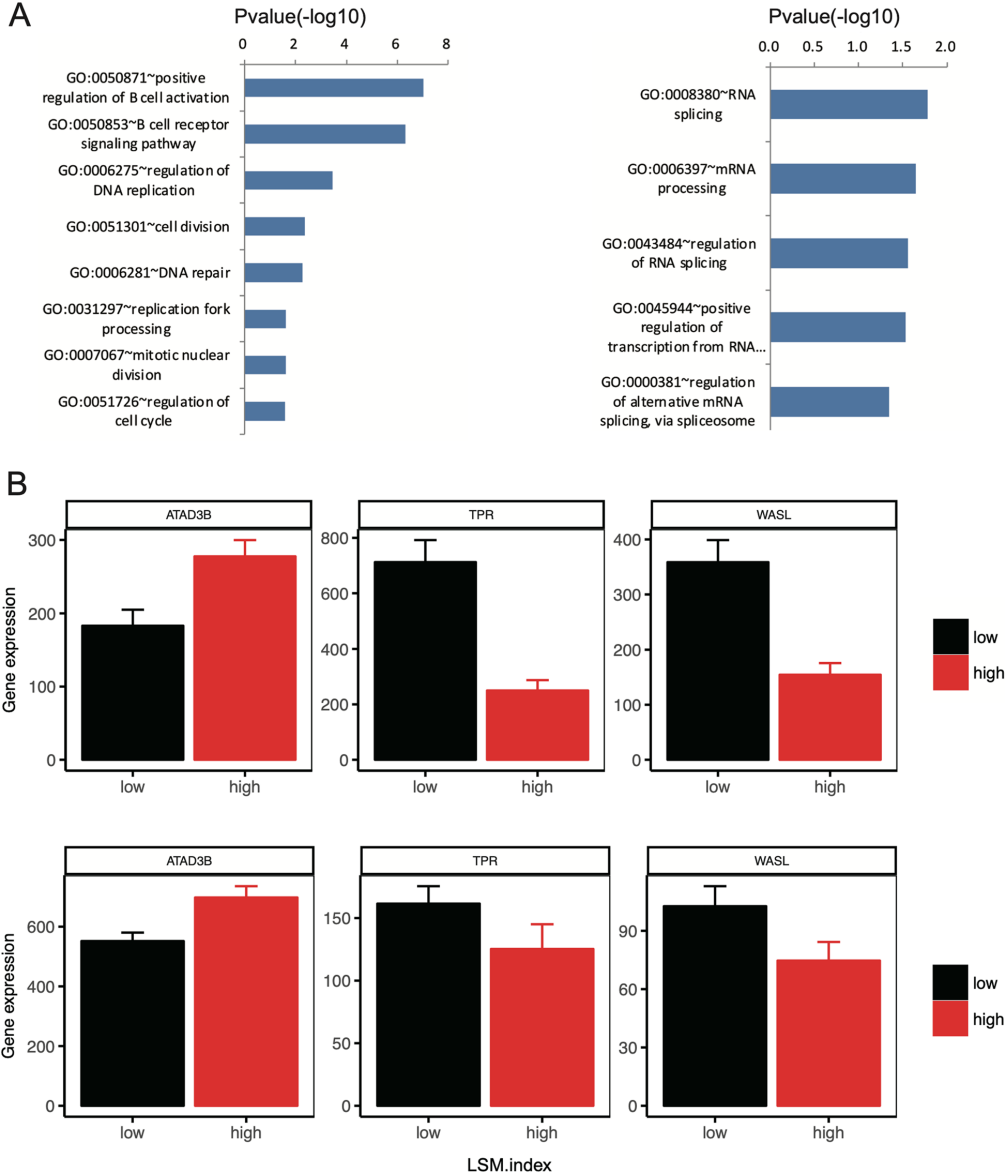
Enriched pathways of differentially expressed genes between *LSM*.index-high and *LSM*.index-low groups in MCL. **A** Bar plot presents the notably enriched positive regulation of cell division pathway (left) and RNA splicing pathway (right). **B** In the cell division pathway, differentially expressed genes between *LSM*.index-high and *LSM*.index-low groups in MCL (mean ± SEM). GSE93291 dataset is shown in the left plot (total 123 samples), while the GSE21452 dataset appears in the right plot (total 64 samples)

We focused on the differential gene expression pathways between the *LSM*.index-high and *LSM*.index-low groups in MCL. The most significantly enriched pathway among all differentially expressed genes was the positive regulation of the B cell activation pathway, followed by the cell division and RNA splicing pathways (Fig. 6A). Among the differentially expressed genes, *ATAD3B*, *TRR*, and *WASL* were identified as being either upregulated or downregulated in the cell division pathway (Fig. 6B). Suppression of *LSM1* expression causes cell cycle arrest in the G2-M phase ^18^. Therefore, *ATAD3B*, *TRR*, and *WASL* may be the target genes regulated by LSM that mediate cell cycle arrest. The differential expression patterns of these genes were also observed in another dataset (GSE21452, 64 samples). These findings suggest that *LSM* genes may regulate the cell division pathway, potentially contributing to poor survival outcomes in patients with MCL.

## Discussion

*LSM1*, also known as “CaSm” (“Cancer-associated Sm-like”), is overexpressed in various cancer, including esophageal, lung, bladder, and prostate cancer. It functions as an oncogene by altering mRNA stability [18, 19]. However, the prognostic significance and biological implications of *LSM* family genes in MCL remain unclear. In this study, we analyzed the expression of *LSM* genes in MCL and found that their dysregulated expression predicts poorer survival outcomes and affects RNA expression across the entire transcriptome.

MCL is one of the rarest types of non-Hodgkin lymphoma and a subtype of B-cell lymphoma that is challenging to treat and is rarely considered curable. The median survival time for patients with MCL ranges from approximately 3 to 6 years. Therefore, identifying new biomarkers to predict survival outcomes in MCL is crucial [26]. The MCL International Prognostic Index (MIPI) is currently the most commonly used prognostic model [27]. It was derived from a cohort of 455 patients with advanced-stage MCL treated in a series of clinical trials in Germany and Europe. The MIPI incorporates the Eastern Cooperative Oncology Group (ECOG) performance status, age, leukocyte count, and lactic dehydrogenase levels and stratifying patients with MLC into three risk groups: low-risk, intermediate-risk, and high-risk [28]. Although various prognostic indicators for MCL have been considered, there is still no universal consensus on forecasting outcomes. KI67 and P53 abnormalities are critical prognostic factors in MCL. High KI67 expression is associated with poor clinical outcomes, and P53 abnormalities often indicate resistance to standard therapies. Prognostic indices such as the MIPI and MIPI-C, which include KI67, are valuable for patient stratification. The MCL35 classification based on RNA expression analysis utilizes genomic and transcriptomic profiling to identify molecular subsets of MCL, thereby providing enhanced prognostic precision and guiding personalized therapeutic strategies [29]. However, these models lack molecular biological predictive features such as gene expression factors. Our analysis revealed that the expression levels of five *LSM* genes were significantly associated with survival in patients with MCL. Additionally, we developed a comprehensive *LSM*.index to predict poor survival outcomes in these patients. The *LSM*.index demonstrated superior predictive power for survival compared to individual LSM proteins (*P* = 3.29E-06).

Notably, more than half of the *LSM* genes (five out of eight) were associated with survival outcomes in patients with MCL (*p* < 0.05, log-rank test). This suggests that *LSM* genes are associated with survival in patients with MCL. Moreover, the *LSM*.index serves as a better predictor of survival, indicating that the imbalanced expression of *LSM* genes may correlate with shorter survival times in these patients. Additionally, we provide three key pieces of evidence to support a strong connection between *LSM* genes and MCL prognosis. First, *LSM* gene expression serves as an effective classifier for MCL. Second, based on the *LSM*.index, we categorized the MCL samples into two groups: *LSM*.index-high and *LSM*.index-low. The *LSM*.index-high group exhibited lower RNA expression levels throughout the transcriptome. Third, in the *LSM*.index-high group, we discovered that the differentially expressed genes were related to the positive regulation of cell division and RNA splicing pathways, which may contribute to poorer survival outcomes in MCL patients.

 Our study shows that *LSM1* is considered to be among the “harmful genes,” with a hazard ratio greater than 1 (*P* = 4.5E-03); conversely, *LSM8* is among the “favorable genes,” with a hazard ratio lower than 1 (*P* = 1.7E-02). The *LSM1–7* and *LSM2–8* complexes mediate cytoplasmic and nuclear mRNA decay, respectively [2–7]. Furthermore, *LSM1* and *LSM8* are the key subunits that differentiate the *LSM1-7* complex from the *LSM2-8* complex [2–7]. Our analysis suggests that *LSM1–7* and *LSM2–8* complexes, which mediate mRNA decay, may play distinct roles in MCL tumorigenesis. Additionally, this study revealed that although *LSM1* was a significant differentiator between prognostic groups, its expression was not significantly different between MCL and reactive lymph nodes. This result suggests that *LSM1* might be more involved in the cellular biological processes underlying malignant transformation in lymphoma rather than serving as a distinguishing biomarker between malignant and reactive proliferation. In contrast, *LSM8* expression was significantly different between MCL and reactive lymph nodes, indicating that *LSM8* may play a more direct role in these pathological conditions. Therefore, *LSM1* is more likely to be a regulatory factor influencing disease progression or prognosis than a direct driver of disease onset. Future studies are required to investigate the specific functions of *LSM1* further to understand its role in lymphomas better.

## Supplementary Information

Supplementary Material 1.

Supplementary Material 2.

## Data Availability

No datasets were generated or analysed during the current study.

## Abbreviations

Cor: Correlation coefficient
GEO: Gene Expression Omnibus
GO: Gene Ontology
MCL: Mantle cell lymphoma
MIPI: MCL International Prognostic Index
