# Supplementary figures and images for: Prognostic significance and biological implications of SM-like genes in mantle cell lymphoma
Source: Blood Res. 2024 Oct 17;59(1):33. doi: 10.1007/s44313-024-00037-3 (PMC11486876; doi:10.1007/s44313-024-00037-3)

# Supplementary Figure 1

A

Group ■ Reactive.Lymph.node ■ MCL

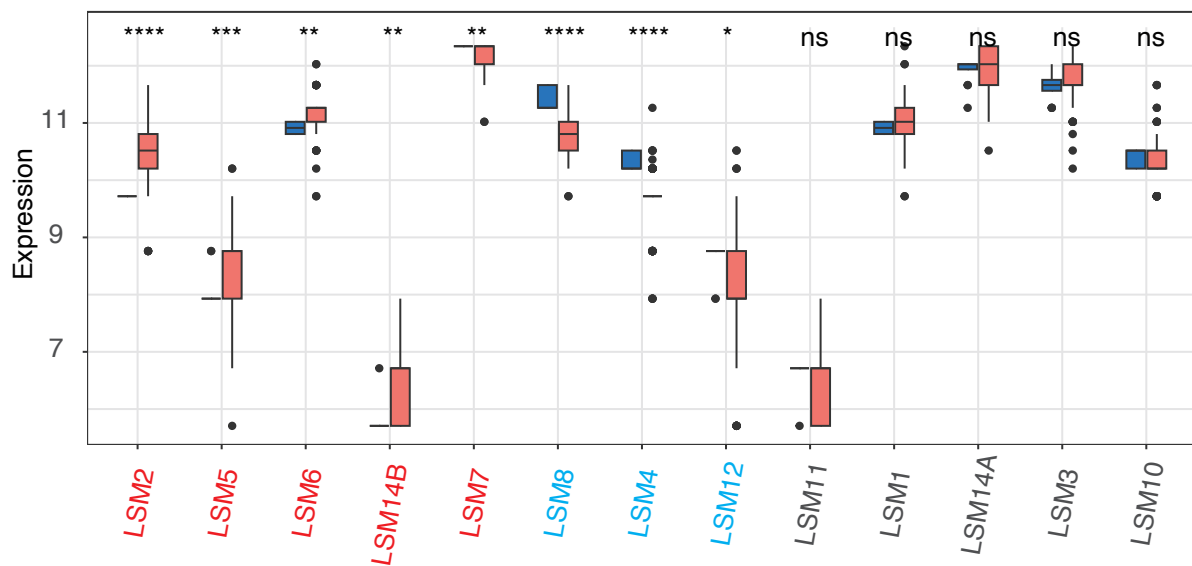

B

Group ■ LSM.index.low ■ LSM.index.high

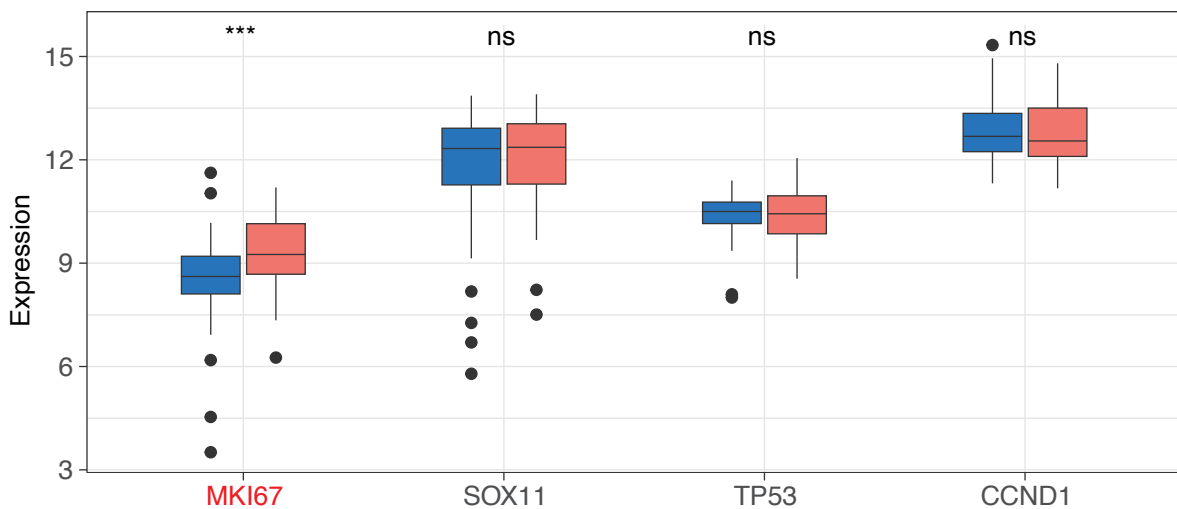

**A**

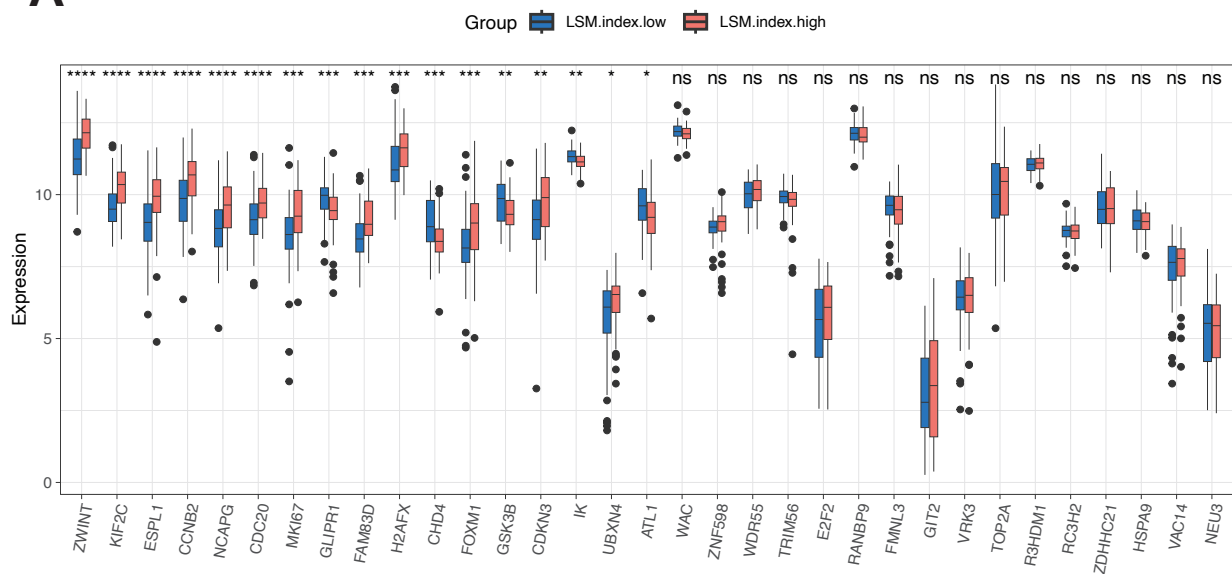

Supplement: Supplementary file 1 — Supplementary Material 1. [file 44313_2024_37_MOESM1_ESM.pdf]
